# Consistent melting behavior induced by Joule heating between Ag microwire and nanowire meshes

**DOI:** 10.1186/1556-276X-9-239

**Published:** 2014-05-15

**Authors:** Kaoru Tsuchiya, Yuan Li, Masumi Saka

**Affiliations:** 1 Department of Nanomechanics, Tohoku University, Aoba 6-6-01, Aramaki, Aoba-ku, Sendai 980-8579, Japan

**Keywords:** Consistent feature, Figure of merit, Joule heating, Melting behavior, Ag microwire/nanowire mesh, Transparent conductive electrode

## Abstract

The melting behavior of an Ag microwire mesh induced by Joule heating was numerically investigated and compared with that of the corresponding Ag nanowire mesh with the same structure but different geometrical and physical properties of the wire itself. According to the relationship of melting current and melting voltage during the melting process, a similar repetitive zigzag pattern in melting behavior was discovered in both meshes. On this basis, a dimensionless parameter defined as figure of merit was proposed to characterize the current-carrying ability of the mesh. The consistent feature of figure of merit in both meshes indicates that the melting behavior of the Ag nanowire mesh can be predicted from the present results of the corresponding Ag microwire mesh with the same structure but made from a different wire (e.g., different size, different material) through simple conversion. The present findings can provide fundamental insight into the reliability analysis on the metallic nanowire mesh-based transparent conductive electrode.

## Background

To meet the requirement of next-generation flexible optoelectronics for both information (e.g., display, electronic reader, touch screen) and energy (e.g., solar cell, window glass), there is growing interest to develop transparent conductive electrodes (TCEs) possessing high optical transmission, good electrical conductivity, and excellent flexibility [1,2]. However, the present common commercial TCE material, i.e., indium tin oxide (ITO), suffers from several major limitations [3-5], such as high cost due to the shortage of indium and poor mechanical stability due to the brittleness. Therefore, it is highly desirable to find a promising alternative which can be used in the forthcoming TCEs [6]. Recently, the network of various nanostructured materials (e.g., carbon nanotube [7,8], graphene [9-11], metallic nanowire [12-20] /nanotrough [21] /honeycomb [22], and the combinations of the above [3,23-25]) has shown great potential for the application in optoelectronic devices such as solar cells [9,16-18] and touch screens [14,20].

Here, our focus is on metallic nanowire mesh (i.e., regular nanowire network) because of its ideal characteristics of low sheet resistance, high optical transparency, and flexible controllability. For example, Kang et al. [16] have fabricated a Cu nanowire mesh electrode on a polyethylene terephthalate (PET) substrate, which shows compatible optical transmittance in the visible wavelength range with commercial ITO-coated PET and offers lower sheet resistance than ITO. Moreover, the short-circuit current and power conversion efficiency of a solar cell with this type of Cu nanowire mesh electrode are comparable to those of the same device using an ITO electrode.

However, realizing the potential benefits of such metallic nanowire mesh in practical optoelectronic devices remains a great challenge because of the lack of reliability analysis. It is known that the pathway of current in a metallic nanowire mesh remains in the nanowire itself, instead of uniform distribution throughout the whole ITO film. Great reduction in current flow area will cause enormous increase in current density and significant rise in temperature due to Joule heating. Therefore, it is believed that the melting induced by Joule heating is a potential threat to the degradation of the metallic nanowire mesh-based TCE, which may cause deterioration of the corresponding optoelectronic devices. In a pioneering experimental report, Khaligh and Goldthorpe [26] have indicated that at a constant current density, a random Ag nanowire network fails after a certain period. Moreover, the network with higher sheet resistance carrying greater current density will fail more easily because of Joule heating. Hereafter, a numerical method has been developed [27] by the present authors to clarify the melting behavior of metallic nanowire mesh due to Joule heating. Using this technique, a repetitive zigzag pattern in the relationship of melting current and melting voltage triggering the melting of the mesh was discovered. It indicates that in real working conditions, a metallic nanowire mesh supplied with current source may experience repetitive unstable (where several wires are melted simultaneously at a constant current/voltage) and stable (where an increment of current/voltage is necessary for melting progression) melting behavior until the mesh is open. However, some of these predicted intrinsic features in the melting of the metallic nanowire mesh would not be detectable because of the difficulty in sample preparation and experimental measurement.

To overcome the above weakness, the relatively easy-to-prepare microwire mesh comes into the sight. One might expect the melting behavior of microwire and nanowire meshes to be similar by assuming that the currents would just scale up. However, metallic nanowire in general displays different properties from microwire because of significant size effect. For example, with decreasing dimension, melting point and thermal conductivity decrease while electrical resistivity increases. Such differences make it difficult to insist on the similarity of the melting behavior for microwire and nanowire meshes, even if both of which have the same structure under the same working conditions.

Herein, to find the intrinsic relationship of the melting behavior between metallic microwire and nanowire meshes, the melting behavior of an Ag microwire mesh was numerically investigated and compared to that of the corresponding Ag nanowire mesh, which has the same mesh structure but different geometrical and physical properties of the wire itself. A similar zigzag pattern was observed in the relationship between melting current and melting voltage of both meshes. Therefore, a dimensionless parameter defined as figure of merit was proposed to indicate the current-carrying ability of the mesh. The consistent figure of merit during the whole melting process of both meshes implies that the melting behavior of the nanowire mesh is predictable from that of the microwire mesh by simple conversion. The present findings provide fundamental insight into the reliability analysis on the metallic nanowire mesh hindered by difficult sample preparation and experimental measurement, which will be helpful to develop ideal metallic nanowire mesh-based TCE with considerable reliability.

## Methods

A previous numerical method [27] was employed to investigate the melting behavior of an Ag microwire mesh and compared with that of the corresponding nanowire mesh which has the same mesh structure (e.g., pitch size, segment number, and boundary conditions) but different geometrical and physical properties of the wire itself (e.g., cross-sectional area, thermal conductivity, electrical resistivity, and melting point).

**Figure 1:**
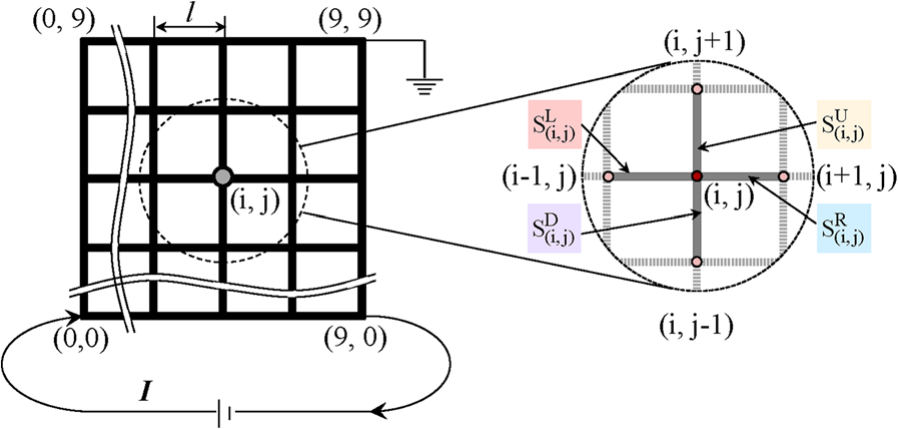
Structure of a wire mesh with size of 10@10 and its electrical boundary conditions.

The mesh structure is illustrated in Figure 1. It is a regular network with 10 columns and 10 rows, which indicates that the mesh size M@N is 10@10. The pitch size *l* is 200 μm, making the mesh area *S* of 3.24 × 10^6^ μm^2^. A mesh node (*i*, *j*) denoted by integral coordinates (0 ≤ *i* ≤ *M* - 1, 0 ≤ *j* ≤ *N* - 1) is the intersection of the (*i* + 1)th column and the (*j* + 1)th row in the mesh. A mesh segment is the wire between two adjacent mesh nodes. For simplicity, the segments on the left, right, downside, and upside of the mesh node (*i*, *j*) are denoted by $S_{\left(i,j\right)}^{L}$, $S_{\left(i,j\right)}^{R}$, $S_{\left(i,j\right)}^{D}$, and $S_{\left(i,j\right)}^{U}$, respectively. Obviously, there are *M* × *N* = 100 mesh nodes and *M*(*N* - 1) + *N*(*M* - 1) = 180 mesh segments.

The electrical boundary conditions are also shown in Figure 1. The load current *I* is input from node (0, 0) and is output from node (9, 0) with zero electrical potential at node (9, 9). Moreover, there is no external input/output current for all the other nodes. For the thermal boundary conditions, the temperature of the peripheral nodes (i.e., (*i*, 0), (0, *j*), (*i*, 9), (9, *j*)) is set at room temperature (RT, *T*_0_ = 300 K), while there is no external input/output heat energy for all the other nodes.

The geometrical and physical properties of the wires are listed in Table 1. Here, *A* is the cross-sectional area calculated from the side length *w* of the wire with the square cross section, *T*_m_ is the melting point, *λ* is the thermal conductivity, and *ρ* is the electrical resistivity with the subscripts ‘0’ and ‘m’ representing the value at *T*_0_ and *T*_m_. Note that *ρ*_m_ [=*ρ*_0_{1 + *α*(*T*_m_ - *T*_0_)}] is calculated by using the temperature coefficient of resistivity *α*. Note that the bulk values of Ag were employed for the microwire, while size effect was taken into account for the nanowire. Because of electron scattering from surface and grain boundaries [28,29], the thinner the wire is, the lower *T*_m_, *λ*, and electrical conductivity (=1/*ρ*) are.

**Table 1:** Geometrical and physical properties of the wires

	**Ag microwire**	**Ag nanowire**	**Al nanowire**
Side length, *w* (μm)	1.000	0.1000	0.1000
Cross-sectional area, *A* (×10^-2^ μm^2^)	100.0	1.000	1.000
Melting point, *T*_m_ (×10^3^ K)	1.234	0.873 [30] (exp.)	0.736 [31] (num.)
Thermal conductivity at RT, λ (×10^-4^ W/μm∙K)	4.200	3.346 [28] (num.)	1.150 [32] (num.)
Electrical resistivity at RT, *ρ*_0_ (×10^-2^ Ω∙μm)	1.590	11.90 [29] (exp.)	6.20 [32] (exp.)
Electrical resistivity at *T*_m_, *ρ*_m_ (×10^-2^ Ω∙μm)	7.200	37.80	17.72

**Figure 2:**
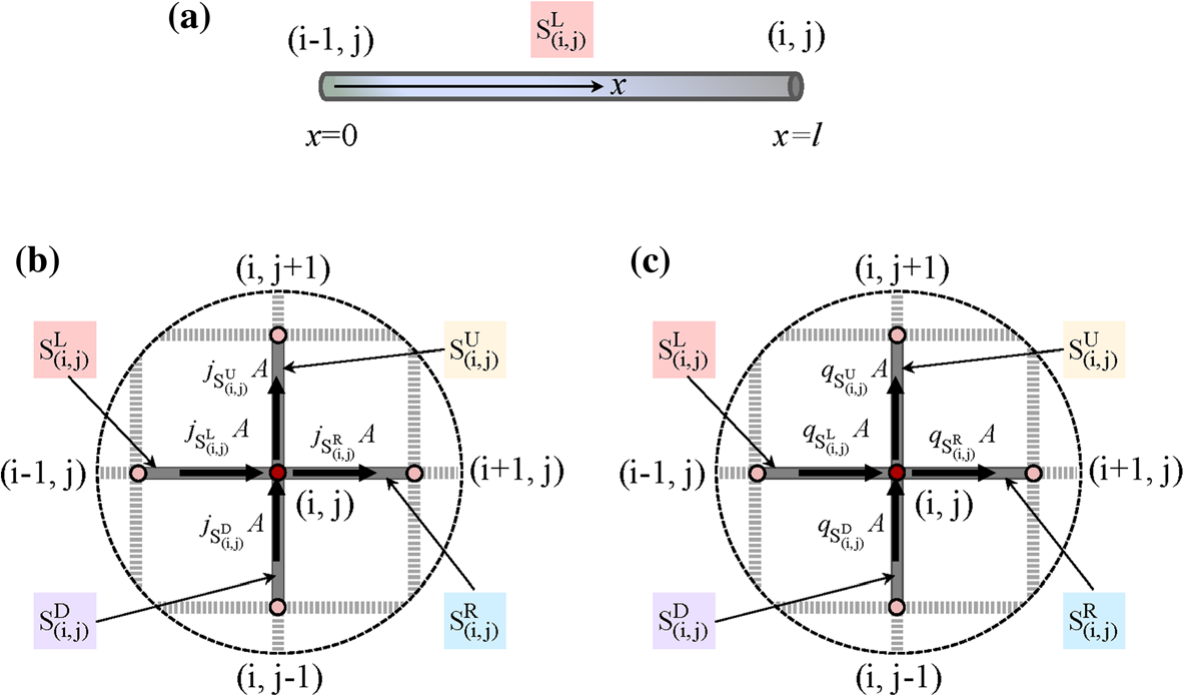
**Theoretical analysis on the electrothermal problem of the wire mesh. (a)** Mesh segment, **(b)** current passing through mesh node (*i*, *j*), and **(c)** heat energy passing through mesh node (*i*, *j*).

To clarify the melting behavior of the mesh, the fundamental theoretical analyses [27] on the corresponding electrothermal problem is summarized in the following. First, as shown in Figure 2a, a horizontal mesh segment (i.e., a wire) $S_{\left(i,j\right)}^{L}$ between node (*i* - 1, *j*) and (*i*, *j*) with an electrically and thermally insulated surface was considered, where the current flows from node (*i* - 1, *j*) to (*i*, *j*). Based on Ohm's law, the current density *j* in the mesh segment can be calculated as

(1) $$j=-\frac{1}{\rho }\frac{d\varphi }{\text{dx}}.$$

Here, *φ* is the electrical potential, and *x* is the axial coordinate in the mesh segment with the direction rightward for horizontal segment and upward for vertical segment. Using Fourier's law, the heat flux *q* in $S_{\left(i,j\right)}^{L}$ can be calculated as

(2) $$q=-\lambda \frac{\text{dT}}{\text{dx}},$$

where *T* is temperature.

By ignoring heat transfer of the mesh to the underlying substrate for simplicity, the heat conduction equation can be given as

(3) $$\lambda \frac{d^{2}T}{dx^{2}}+\rho j^{2}=0.$$

Assuming that the temperatures of nodes (*i* - 1, *j*) and (*i*, *j*) are *T*_(*i*-1.*j*)_ (*x* = 0) and *T*_(*i*,*j*)_ (*x* = *l*), temperature distribution in the mesh segment $S_{\left(i,j\right)}^{L}$ can be obtained by solving Equation 3 as

(4) $$T=-\frac{\rho }{2\lambda }j^{2}x^{2}+\left\{\frac{T_{\left(i,j\right)}-T_{\left(i‐1,j\right)}}{l}+\frac{\rho l}{2\lambda }j^{2}\right\}x+T_{\left(i‐1,j\right)}.$$

Note that in the present simulation, *ρ*_m_ was used for *ρ* to approximate real condition neglecting the effect of the temperature dependence of electrical resistivity.

Second, as shown in Figure 2b,c, the current and heat energy passing through a mesh node (*i*, *j*) with four adjacent nodes were considered. In Figure 2b, the current is assumed to flow rightward in the horizontal direction and upward in the vertical direction. According to Kirchhoff's current law, we have

(5) $$I_{\text{external}}+I_{\text{internal}}=0.$$

Here, *I*_external_ is the external input/output current at node (*i*, *j*), and *I*_internal_ is the sum of internal currents flowing through the node (*i*, *j*) from its four adjacent nodes. By assuming that the current flowing into the node is positive and the current flowing out of the node is negative, we can obtain

(6) $$I_{\text{internal}}=\left\{j_{S_{\left(i,j\right)}^{L}}-j_{S_{\left(i,j\right)}^{R}}+j_{S_{\left(i,j\right)}^{D}}-j_{S_{\left(i,j\right)}^{U}}\right\}A,$$

where the subscript of *j* denotes the corresponding mesh segment. Taking into account a system of linear equations for the node (*i*, *j*) composed of Equations 1, 5, and 6, the current density in any mesh segment can be obtained. Similarly, according to the law of the conservation of heat energy, we have

(7) $$Q_{\text{external}}+Q_{\text{internal}}=0.$$

Here, *Q*_external_ is the external input/output heat energy at node (*i*, *j*), and *Q*_internal_ is the sum of the internal heat energy transferred to node (*i*, *j*) from its four adjacent nodes. In Figure 2c, by assuming that the incoming heat energy is positive and the outgoing heat energy is negative, we have

(8) $$Q_{\text{internal}}=\left\{q_{S_{\left(i,j\right)}^{L}}-q_{S_{\left(i,j\right)}^{R}}+q_{S_{\left(i,j\right)}^{D}}-q_{S_{\left(i,j\right)}^{U}}\right\}A.$$

Taking into account a system of linear equations for the node (*i*, *j*) composed of Equations 2, 7, and 8, the temperature at any mesh node can be obtained. Finally, by substituting the above obtained current density in any mesh segment and temperature at any mesh node into Equation 4, the temperature distribution in any mesh segment can be monitored.

A synopsis of the corresponding computational algorithm [27] is provided as below. Initially, a small value is assigned to the input current *I*. The corresponding maximum temperature in the mesh *T*_max_ can be identified, which rises with the increasing *I*. By gradually increasing *I* with increment Δ*I* to make *T*_max_ reach *T*_m_, the first mesh segment melts and breaks from an arbitrary small force occurring in actual operation (e.g., vibration). At that time, the input current and the voltage between node (0, 0) and node (9, 0) are recorded as melting current *I*_m_ and melting voltage *V*_m_. The corresponding resistance *R*_m_ of the mesh can be calculated by dividing *V*_m_ by *I*_m_. It should be noted that Δ*I* must be small enough so that melting segment can melt one by one as far as possible. Subsequently, an ultra-small value is assigned to the cross-sectional area of the first melted mesh segment in order to approximate zero. The pathway of the current and heat in the mesh is therefore renewed. By repeating the aforementioned process, the current triggering the melting of mesh segment one by one can be obtained until the mesh becomes open. Therefore, the relationship between *I*_m_ and *V*_m_ as well as the variation of *R*_m_ with the number *n*_b_ of the broken mesh segments can be obtained over the entire melting process of the mesh.

## Results and discussion

### Melting behavior of the Ag microwire mesh

**Figure 3:**
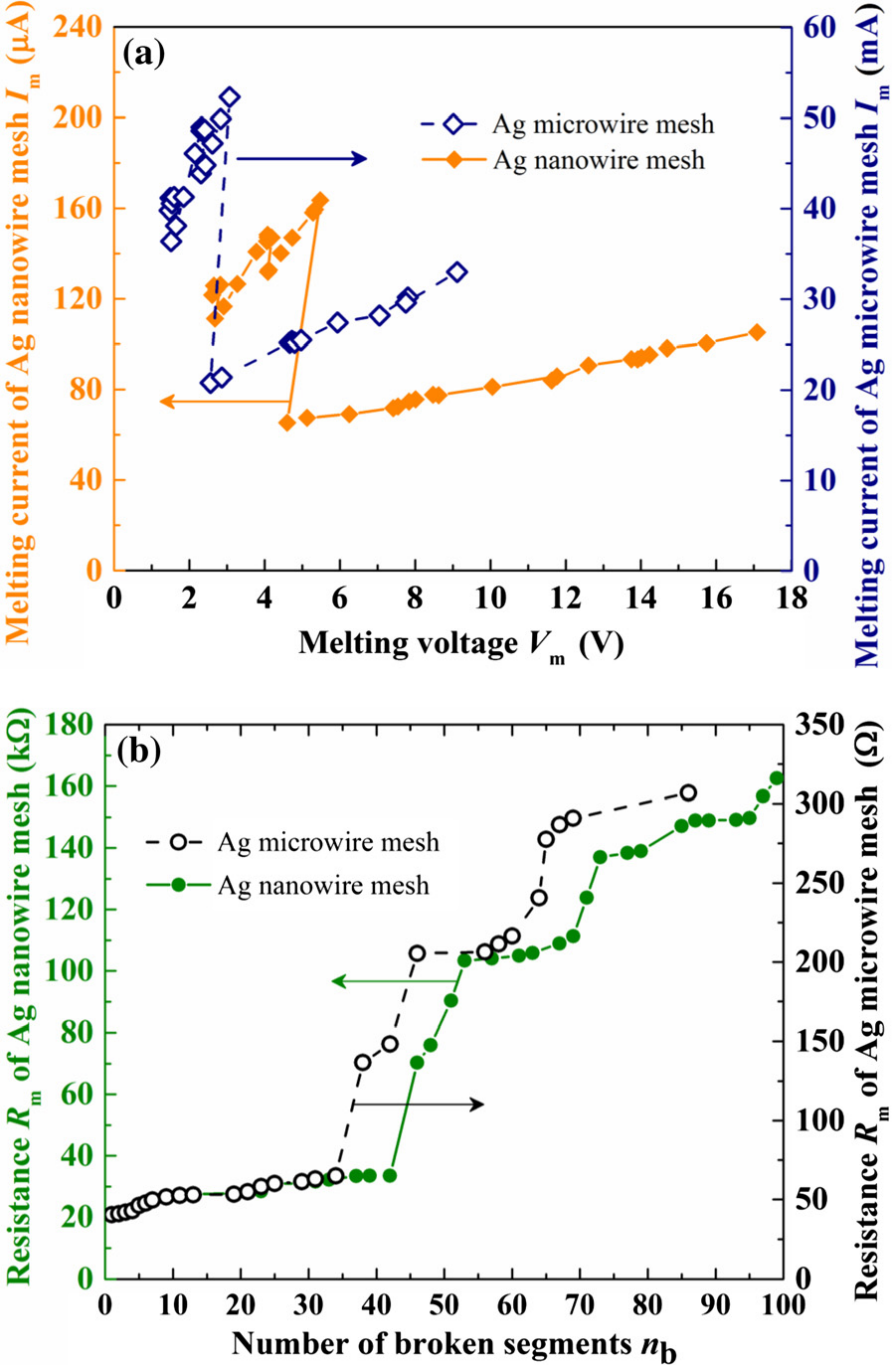
**Comparison of melting process for both meshes. (a)** The relationship between *I*_m_ and *V*_m_, and **(b)** the variation of *R*_m_ with *n*_*b*_.

As shown in Figure 3a,b, the obtained relationship of melting current *I*_m_ and melting voltage *V*_m_ as well as the variation of mesh resistance *R*_m_ with the number *n*_b_ of broken mesh segments during the entire melting process of the Ag microwire mesh is compared with those of the corresponding Ag nanowire mesh, respectively.

Obviously, a repetitive zigzag pattern is observed in the relationship of *I*_m_ and *V*_m_ in the Ag microwire mesh, which demonstrates the repetition of three different trends: increase of both *I*_m_ and *V*_m_, decrease of both *I*_m_ and *V*_m_, and decrease of *I*_m_ but increase of *V*_m_. Such pattern in the melting behavior of Ag microwire mesh is similar with that of the corresponding Ag nanowire mesh [27]. Note that the microwire mesh has higher *I*_m_ but lower *V*_m_ than the nanowire mesh, because the microwire has larger cross-sectional area and lower resistivity (see Table 1) and therefore lower electrical resistance (see Figure 3b) than the nanowire.

**Figure 4:**
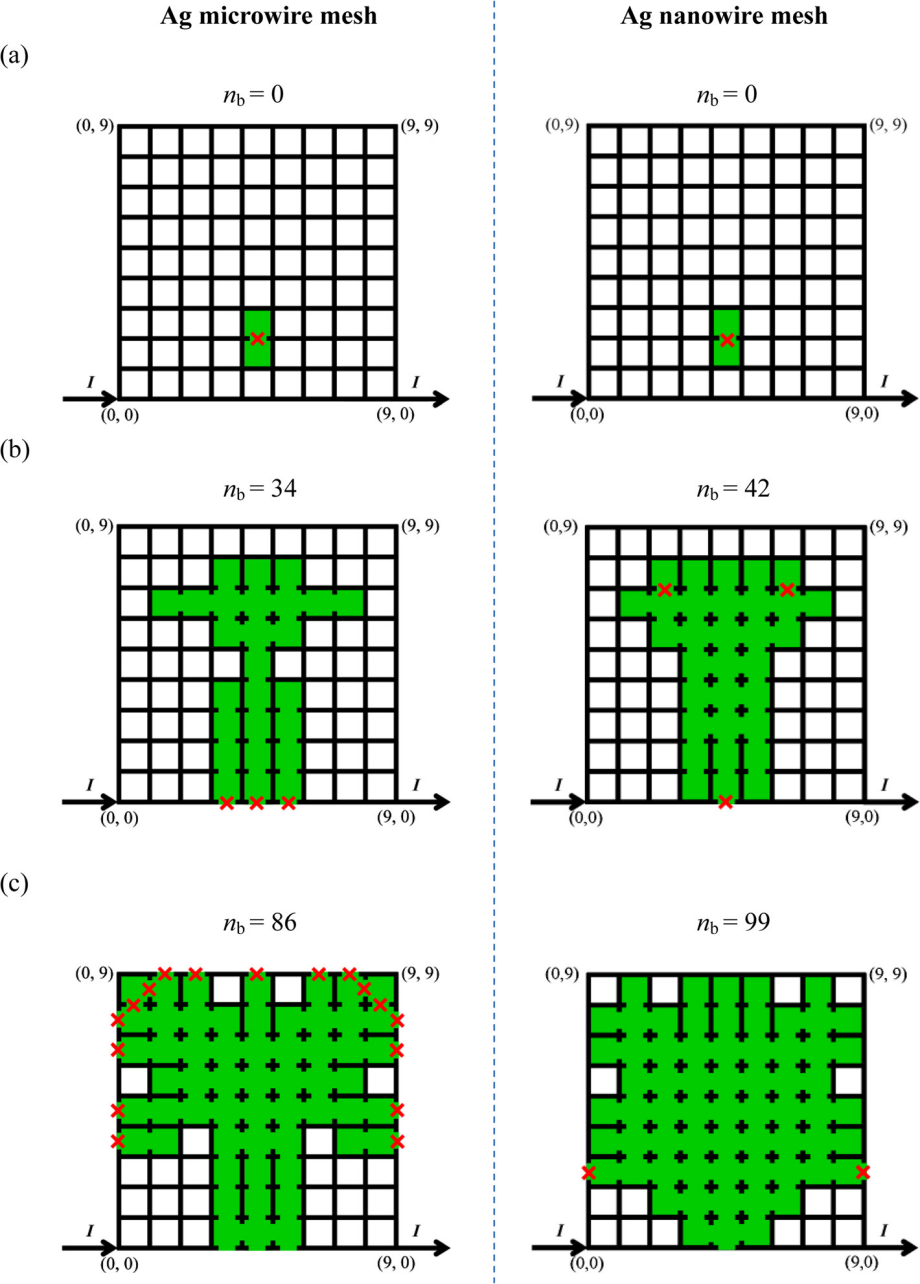
**Mesh structures at three special moments in the melting process of both meshes. (a)** The starting moment, **(b)** the moment with the maximum current (i.e., sudden fall of current), and **(c)** the ending moment.

During the melting process, the symmetrical mesh structures at three special moments for both meshes are compared in Figure 4. The difference in the melting pathway of both meshes can be attributed to the different ∆*I* for monitoring the melting of mesh segment, which are 0.1 mA for the Ag microwire mesh and 0.1 μA for the Ag nanowire mesh. Note that such difference can be removed by employing much smaller ∆*I* for the Ag microwire mesh at the expense of increasing computational cost.

Moreover, from the present simulation results, it is believed that under constant current density (i.e., current-controlled current source), electric breakdown of the mesh will never happen as long as the load current *I* does not reach the maximum value of *I*_m_ (i.e., *I*_mC_) even if several mesh segments melt. This point is quite different from the reported electrical failure of a random Ag nanowire network [26] under constant current density after a certain current stressing period. Such difference between experiments and present simulations also implies that the electrical failure in real Ag nanowire mesh should be the synergy of Joule heating and some other possible causes, such as corrosion by sulfur, atomic diffusion in the nanowire itself, and Rayleigh instability [26].

### Proposal of figure of merit *Z*

To explore the intrinsic characteristics of the melting behavior of metallic microwire and nanowire meshes, it would be helpful to find a common parameter which is independent of geometrical and physical properties of the mesh. In order to deduce such a parameter, let us consider a simple model of a wire subjected to a constant current as shown in Figure 2a. By neglecting the difference between *T*_(*i*,*j*)_ and *T*_(*i*-1,*j*)_ for simple approximation, the following equation can be easily obtained from Equation 4:

(9) $$T_{C}-T_{\left(i,j\right)}=j^{2}\frac{\rho }{8\lambda }l^{2},$$

where *T*_C_ is the maximum temperature occurring in the center of the wire with *x* = *l*/2. It indicates that *j*^2^*l*^2^(*ρ*/*λ*)/(*T*_C_ - *T*_(*i*,*j*)_) is independent of geometrical and physical properties of the wire.

**Figure 5:**
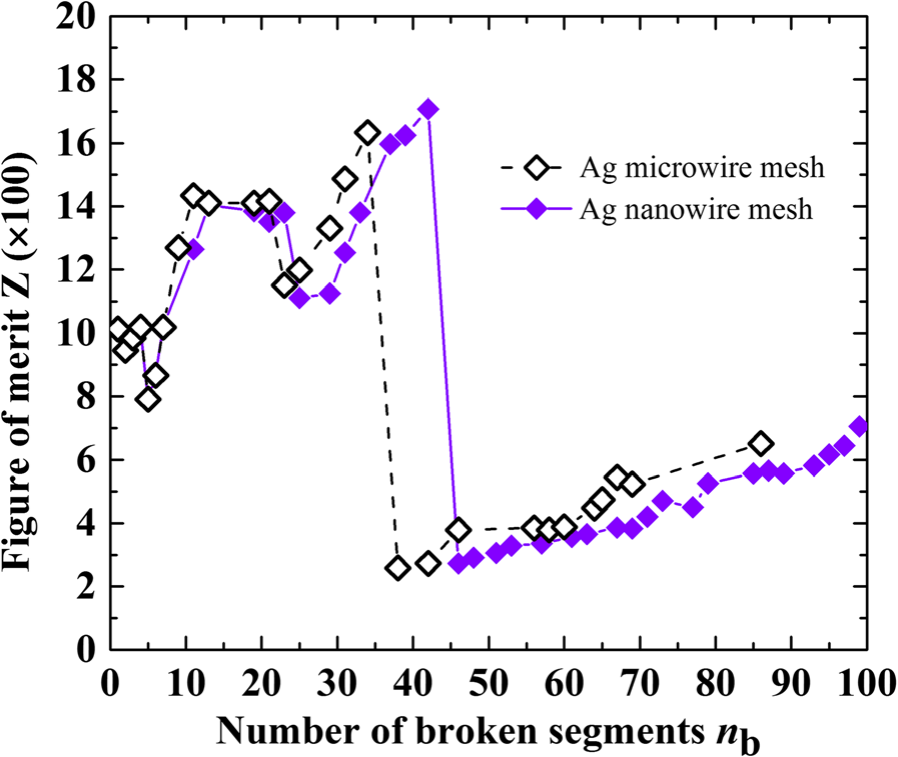
**Variation of *Z *** **with *n***_**b **_**in the melting process of both meshes.**

Based on the above consideration, the following dimensionless parameter *Z* was proposed as figure of merit of the mesh:

(10) $$Z={\left(\frac{I_{m}}{A}\right)}^{2}\frac{\rho }{\lambda }\frac{S}{T_{m}-T_{0}},$$

which indicates the current-carrying ability of the mesh. The variation of calculated *Z* during the melting process is shown in Figure 5, which was developed from the numerical results in Figure 3. Note that the maximum value of *Z* (i.e., *Z*_C_) corresponding to the maximum value of *I*_m_ (i.e., *I*_mC_) characterizes the current-carrying capacity of the mesh, at which the mesh equipped with current-controlled current source will melt until open. Reasonably consistent behavior of *Z* in both meshes can be clearly observed in Figure 5. The minor difference can be attributed to the different melting pathways (see Figure 4), which can be removed by employing much smaller Δ*I* for the microwire mesh with sacrifice of computational cost.

Generally, for the same material, *T*_m_, *ρ*, *λ*, and *A* are dependent on wire size, while *S* is dependent on mesh structure. For a given mesh structure with a known *S*, the smaller *A* results in smaller *T*_m_ and *λ* but larger *ρ*, and therefore smaller *I*_m_ according to Equation 10. This point is the same with the above numerical results where the *I*_m_ of the microwire mesh is significantly higher than that of the nanowire mesh (see Figure 3a).

**Figure 6:**
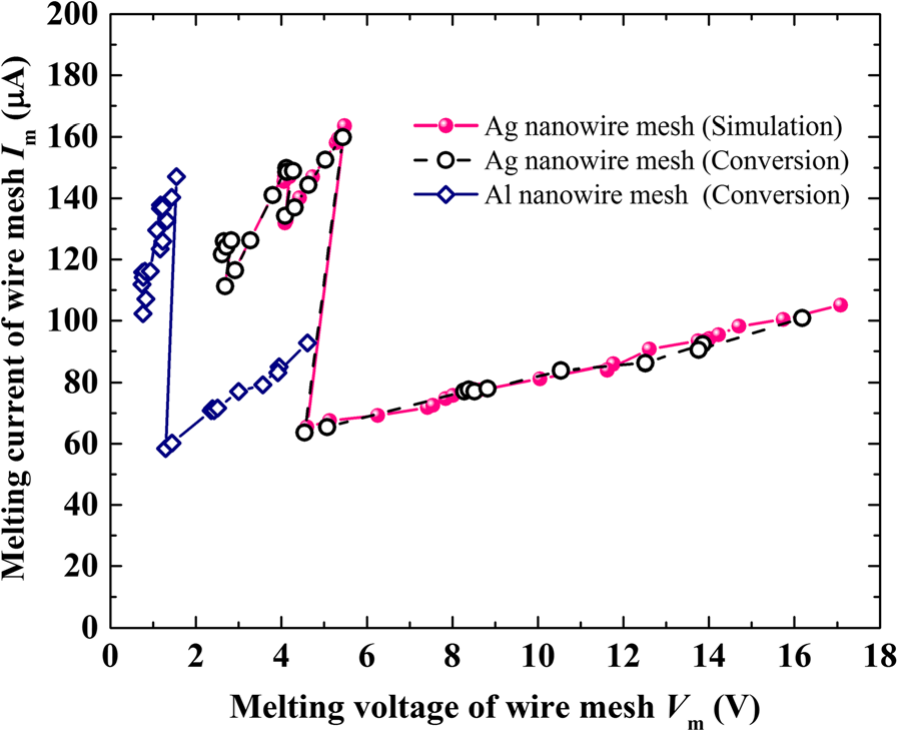
Predicted melting behavior of Ag and Al nanowire meshes by conversion.

Therefore, it is expected that the obtained melting behavior of the microwire mesh can be used to predict that of the wire mesh with same structure at the same working condition even if made from a different wire (i.e., different size, different material) through simple conversion with the known *Z*. Taking the Ag nanowire mesh as an example, the conversion process is summarized here. First, the melting current *I*_m_ for the nanowire mesh can be calculated from Equation 10 with the known *Z*. Second, the variation of the *R*_m_ for nanowire mesh can be calculated from that for the microwire mesh in Figure 3b as

(11) $$R_{m}\left|{}_{\text{NW}}\right)=R_{m}\left|{}_{\text{MW}}\right)\times \left(\frac{\rho_{m}}{A}\right)\left|{}_{\text{NW}}\right)\times \left(\frac{A}{\rho_{m}}\right)\left|{}_{\text{MW}}\right),$$

because of the same melting process. Note that ‘|_NW_’ and ‘|_MW_’ indicate the case for the Ag nanowire mesh and Ag microwire mesh, respectively. Third, the variation of *V*_m_ for the Ag nanowire mesh can be calculated by multiplying the obtained *R*_m_ and *I*_m_ from the above two steps. The predicted melting behavior of the Ag nanowire mesh derived from the above indirect conversion is shown in Figure 6, which indicates good agreement with that obtained from direct numerical simulation, and therefore validates the feasibility of the present conversion method. Figure 6 also gives the predicted melting behavior of the Al nanowire mesh with the same structure through indirect conversion. Obviously, the melting behavior of the mesh is largely dependent on the physical properties of the wire itself.

It should be noted that the present boundary conditions and mesh structure are only one example. Certainly, boundary conditions and mesh structure will have great effect on the melting behavior of the wire mesh as well as physical properties of the wire itself. However, the consistent feature in the melting behavior among the wire meshes with the same structure under the same boundary conditions will not change. Therefore, the present findings can provide meaningful insight for the experimental investigation on the reliability of the metallic nanowire mesh-based TCE.

## Conclusions

In this work, the melting behavior of an Ag microwire mesh induced by Joule heating was numerically investigated and compared with that of the corresponding Ag nanowire mesh with the same mesh structure but different geometrical and physical properties of the wire itself. The repetitive zigzag pattern in the relationship of melting current and melting voltage during the melting process in the Ag microwire mesh was found to be similar with that of the Ag nanowire mesh. A dimensionless parameter *Z* was proposed as figure of merit to characterize the current-carrying ability of the mesh. The consistent behavior of figure of merit in both meshes indicates that the known *Z* and the melting behavior of the Ag microwire mesh can be used to predict the melting behavior of the nanowire mesh even with different materials (e.g., Ag nanowire mesh, Al nanowire mesh), which is hindered by the cost of sample preparation and the difficult control of ultra-low current stressing in experiments. The present findings indicate great insight for reliability analysis on the metallic nanowire mesh-based TCE, which will be beneficial to improve the performance of the corresponding optoelectronic devices.

## Abbreviations

ITO: indium tin oxide; PET: polyethylene terephthalate; RT: room temperature; TCE: transparent conductive electrode.

## Competing interests

The authors declare that they have no competing interests.

## Authors’ contributions

KT carried out the numerical analysis and drafted the manuscript. YL and MS conceived the study, participated in its design, and helped to finalize the manuscript. All authors read and approved the final manuscript.
